# Intestinal APCs of the endogenous nanomineral pathway fail to express PD-L1 in Crohn’s disease

**DOI:** 10.1038/srep26747

**Published:** 2016-05-26

**Authors:** Jack Robertson, Carolin T. Haas, Laetitia C. Pele, Tom P. Monie, Charles Charalambos, Miles Parkes, Rachel E. Hewitt, Jonathan J. Powell

**Affiliations:** 1 Medical Research Council Human Nutrition Research, Elsie Widdowson Laboratory, 120 Fulbourn Road, Cambridge CB1 9NL, UK; 2 Department of Medicine, University of Cambridge School of Clinical Medicine, Cambridge Biomedical Campus, Cambridge, United Kingdom

## Abstract

Crohn’s disease is a chronic inflammatory condition most commonly affecting the ileum and colon. The aetiology of Crohn’s disease is complex and may include defects in peptidoglycan recognition, and/or failures in the establishment of intestinal tolerance. We have recently described a novel constitutive endogenous delivery system for the translocation of nanomineral-antigen-peptidoglycan (NAP) conjugates to antigen presenting cells (APCs) in intestinal lymphoid patches. In mice NAP conjugate delivery to APCs results in high surface expression of the immuno-modulatory molecule programmed death receptor ligand 1 (PD-L1). Here we report that NAP conjugate positive APCs in human ileal tissues from individuals with ulcerative colitis and intestinal carcinomas, also have high expression of PD-L1. However, NAP-conjugate positive APCs in intestinal tissue from patients with Crohn’s disease show selective failure in PD-L1 expression. Therefore, in Crohn’s disease intestinal antigen taken up by lymphoid patch APCs will be presented without PD-L1 induced tolerogenic signalling, perhaps initiating disease.

Crohn’s disease is a chronic and debilitating inflammatory condition of the ileum and colon that afflicts a growing proportion of the Western population. The precise cause of Crohn’s disease is unclear but dietary, lifestyle, genetic and pathogen related factors have all been proposed to be important^1^. Various genetic polymorphisms serve as risk factors for the development of Crohn’s disease, with specific mutations in the innate immune gene *NOD2* presenting the strongest single genetic risk factor^2^. How *NOD2* mutation predisposes to disease is uncertain. It may involve aberrant signalling due to a failure to recognise peptidoglycan, defects in autophagy, disruption of other immune pathways, or alteration of the intestinal microbiota^3^,^4^,^5^.

Recently, we showed that Microfold (M) cells in murine ileal lymphoid patches are portals for the entry of endogenous luminally-formed nanomineral particles^6^. These nanomineral particles are composed of amorphous magnesium-substituted calcium phosphate templated around macromolecular luminal constituents, notably protein antigen and bacterial peptidoglycan, creating a nanomineral-antigen-peptidoglycan (NAP) conjugate. Once chaperoned across M cells, the luminally-derived peptidoglycan and protein antigen, still encased in their nanomineral shell, reach underlying APCs of the sub-epithelial dome where upon the nanomineral appears to readily release its peptidoglycan and protein cargo^6^. The NAP pathway ensures the safe chaperoning of cargo macromolecules from lumen to APC and that cells receiving protein antigen are also the ones receiving peptidoglycan. This is particularly important as peptidoglycan, delivered in this fashion, provides a potent signal for APC surface expression of the immuno-inhibitory, co-regulatory molecule PD-L1^6^,^7^. Indeed, in mice defective in intracellular peptidoglycan recognition there appears to be no expression of PD-L1 on intestinal APCs of the NAP pathway. In contrast, the same APCs of wild type mice are PD-L1^hi ^6.

PD-L1 provides a crucial immuno-inhibitory function. It is central to the development of regulatory T cells, via interaction with its receptor PD-1, and is normally upregulated during inflammation to restrain tissue damage^8^. In the intestines PD-1 is usually expressed on activated T-cells in germinal centres of the Peyer’s Patches, whereas PD-L1 is found on macrophages and dendritic cells in the sub-epithelial dome^9^, as well as intestinal epithelial cells^10^. Physiological expression of PD-L1 has been shown to prevent antigen-induced inflammatory responses of the intestine^11^,^12^,^13^. Consequently, selective PD-L1 upregulation on APCs of the NAP pathway may ensure that luminally-derived antigen is presented in a tolerogenic context to minimise intestinal inflammation. Given the above, and particularly the inability of *Nod1* and *Nod2* deficient mice to express PD-L1 on APCs in the NAP pathway^6^, we asked whether irregularities in PD-L1 expression could underpin the development and pathogenesis of Crohn’s disease. Analysis of ileal tissue samples revealed a dramatic cell selective defect in PD-L1 expression in Crohn’s disease. Specifically, nanomineral positive APCs of the NAP pathway almost ubiquitously failed to express PD-L1 in samples from Crohn’s disease patients, but showed normal PD-L1 expression in controls. Consequently it is probable that in Crohn’s disease the majority of NAP-derived antigens, whether bacteria-derived or other luminal molecules, are not presented in a tolerogenic manner and thereby could contribute to the development and exacerbation of an inflammatory response.

## Results and Discussion

### PD-L1 and Crohn’s disease

Regulated expression of PD-L1 is critical for the control of intestinal inflammation^12^,^13^. We have previously shown that peptidoglycan can upregulate PD-L1 expression^7^ and that mice deficient in *Nod1* and *Nod2* (cytoplasmic receptors for peptidoglycan) display defective PD-L1 expression in their intestinal APCs^6^. In addition, Crohn’s disease associated polymorphisms, including in *NOD2*, disrupt peptidoglycan recognition^7^,^14^. We therefore hypothesised that defects in the delivery or recognition of peptidoglycan to APCs in the intestinal tract would result in aberrant PD-L1 expression and underpin the development of Crohn’s disease.

A comprehensive review of publically available *CD274* gene expression data (*CD274* encodes the PD-L1 protein) did not provide evidence of global changes in *CD274* expression levels between Crohn’s disease and control intestinal tissue (Supplementary Figure 1 and Supplementary Tables 1–3). Only two studies, both looking at inflamed adult colonic Crohn’s disease tissues, identified any significant change in *CD274* expression and in both cases expression was upregulated in the Crohn’s disease tissue (Supplementary Figure 1)^15^,^16^. Such gene expression data does not, however, discriminate at the level of the individual cell, leaving open the possibility that specific cell types within the ileum or colon could display greater disparity in either *CD274* expression, or PD-L1 surface expression. Indeed slightly enhanced PD-L1 protein expression in epithelial cells and in lamina propria mononuclear cells has been reported in Crohn’s disease albeit without widespread validation^17^,^18^.

### PD-L1 expression is detectable in Crohn’s disease ileal tissue

**Figure 1:**
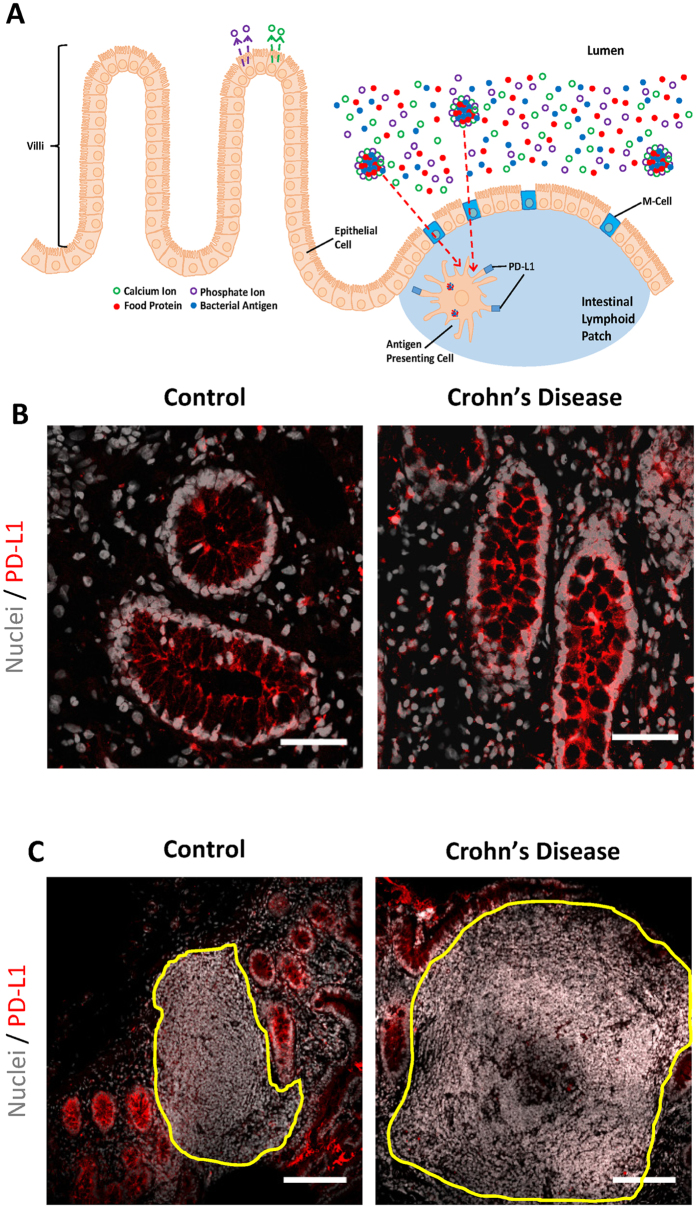
PD-L1 expression is detectable in both Crohn’s disease and control ileal tissue. (**A**) Schematic representation of the cellular organisation of the ileal region. The translocation of luminal antigens to APCs via the nanomineral-antigen-peptidoglycan (NAP) pathway is outlined. In brief, calcium and phosphate ions are secreted from epithelial cells back into the intestinal lumen where they mix with luminal molecules and subsequently precipitate forming nanoparticles which trap small quantities of food proteins and bacterial antigens. The resulting NAP conjugates are translocated from the lumen to the mucosa through M-cells and chaperoned APCs for uptake. Upon digestion of NAPs, peptidoglycan-induced PD-L1 is expressed on the APC surface and contributes to immune tolerance towards intestinal antigens. Typical confocal micrographs showing PD-L1 expression (red) in (**B**) epithelial cells and (**C**) ileal lymphoid patches from Crohn’s disease and control tissue sections. The ileal lymphoid patch is outlined in yellow. Scale bars are 50 μm (**B**) and 150 μm (**C**).

The ileum is the primary target for inflammation in the majority of cases of Crohn’s disease and possesses a complex cellular micro-environment with a carefully defined stratification of cell populations (Fig. 1A). To address potential cell-specific changes in PD-L1 surface expression we studied fresh human ileal tissue sections that had been snap-frozen from patients with and without Crohn’s disease. PD-L1 staining was detected in ileal epithelial cells by confocal microscopy irrespective of disease status (Fig. 1B). Some PD-L1 expression was also detectable in ileal lymphoid patches, showing a low to moderate level of expression in all samples (Fig. 1C). These findings corroborate the earlier gene analysis data showing that, as a whole, there is no lack of PD-L1 expression in Crohn’s disease gut tissue.

### Nanomineral positive cells in Crohn’s disease tissues show a specific defect in PD-L1 expression

**Figure 2:**
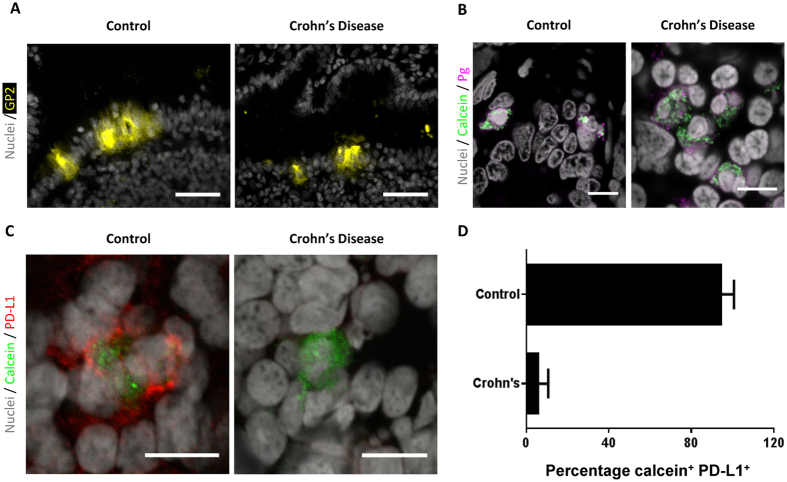
APCs in the NAP pathway show defective PD-L1 expression in Crohn’s disease. (**A**) Representative fluorescent micrographs showing M cells (identified by glycoprotein 2 staining; yellow) in the epithelium overlaying lymphoid patches in Crohn’s disease and control sections. Scale bars are 50 μm. (**B**) Typical confocal micrographs demonstrating the presence of peptidoglycan (Pg; magenta) in nanomineral positive cells (identified by calcein; green) in both Crohn’s disease and control sections. Scale bars are 10 μm. (**C**) Example confocal micrographs of Crohn’s disease and control ileal lymphoid patch cells showing that APCs from control tissue are both nanomineral (calcein-stained, green) and PD-L1 (red) positive, but Crohn’s disease associated APCs are nanomineral positive and PD-L1 negative. Scale bars are 10 μm. (**D**) Percentage of nanomineral (calcein-stained) and PD-L1 positive cells in control and Crohn’s disease tissue (Crohn’s disease *versus* control: p < 0.0004 using the Mann-Whitney test). Data are shown as mean + SD. In all cases samples were tested from 6 independent tissues for Crohn’s disease and 9 independent tissues for control tissues. In total 177 calcein (nanomineral) positive cells were identified in the Crohn’s disease samples, and 191 calcein (nanomineral) positive cells in the control tissues.

Intestinal lymphoid patches are a key site of intestinal antigen uptake in which M cells provide a specialised antigen transportation function^19^. They also provide a focal point for aphthous ulcer-like lesion formation during the earliest microscopic signs of Crohn’s disease^20^. Staining for glycoprotein 2, an M cell specific surface marker^21^, confirmed the presence of M cells in the epithelium of the ileal lymphoid patches in both control and Crohn’s disease samples (Fig. 2A). M cells have recently been shown to be critical for the translocation of NAP-conjugates to underlying APCs^6^. This pathway remains functional in Crohn’s disease, as peptidoglycan co-localised with nanomineral positive cells (identified by calcein staining) are present in both control and Crohn’s disease tissues (Fig. 2B).

**Figure 3:**
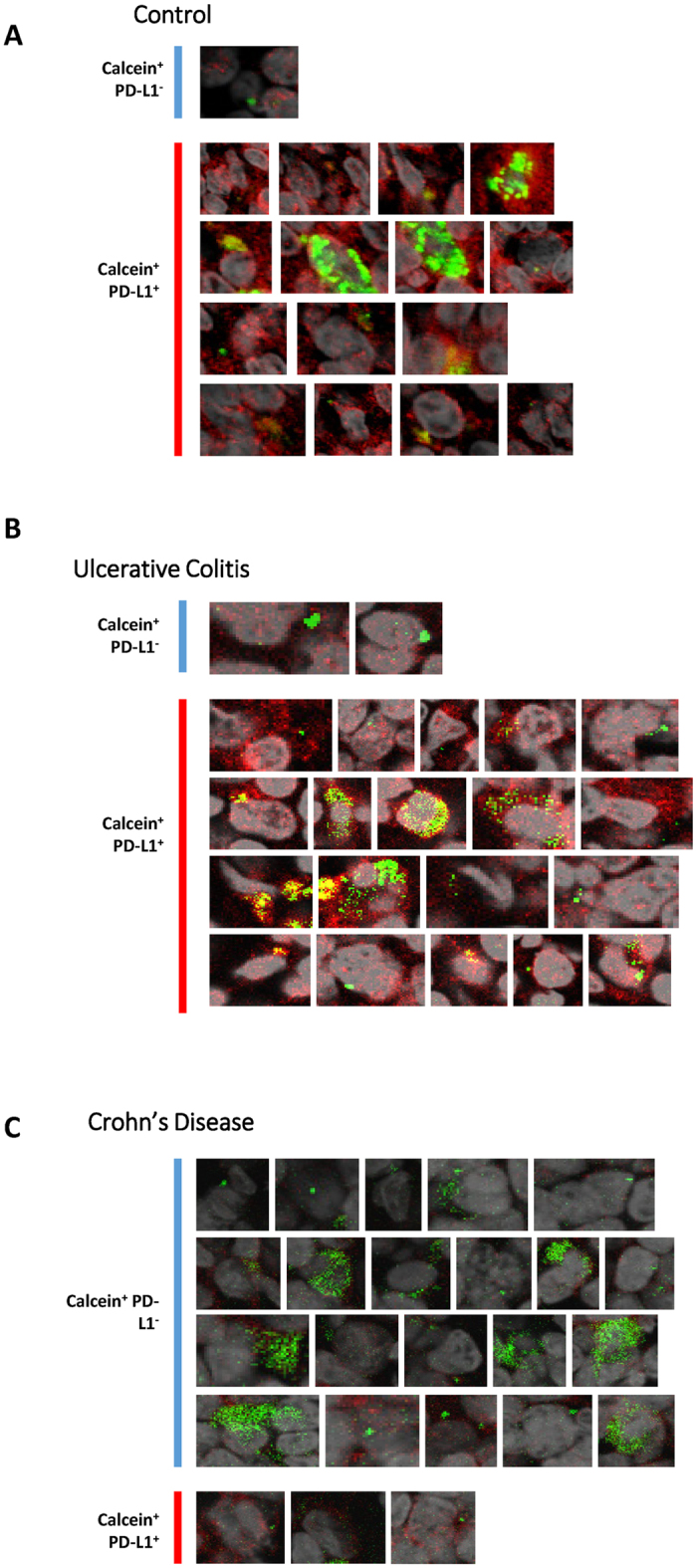
Defective PD-L1 expression is limited to APCs from Crohn’s disease and is not observed in ulcerative colitis. Nanomineral positive cells (green) are only defective for PD-L1 (red) expression in Crohn’s disease. For each section all nanomineral (calcein) positive cells from the 63X micrograph were selected and expanded. Each cell was individually analysed and characterised as PD-L1 negative (Calcein^+^ PD-L1^−^) or PD-L1 positive (Calcein^+^ PD-L1^+^) as described in Materials and Methods. Panels (**A**) control tumour section, (**B**) control ulcerative colitis tissue section and (**C**) Crohn’s disease tissue section are representative of the 9 and 6 tissue samples used for control and Crohn’s disease samples respectively. In each panel all identified calcein (nanomineral) positive cells are shown, regardless of their PD-L1 expression status.

Interestingly, nanomineral positive cells of ileal lymphoid patches (i.e. cells involved in the NAP pathway^6^) showed a dramatic difference in PD-L1 expression between Crohn’s disease and control ileum (Fig. 2C). Control nanomineral positive cells were almost always positive for PD-L1 expression, whereas nanomineral positive cells from Crohn’s disease tissue were almost always negative for PD-L1 (Fig. 2C,D). To verify this observation we manually identified all nanomineral positive cells in our tissue samples and confirmed their PD-L1 status. A cell was defined as being both nanomineral and PD-L1 positive if discrete PD-L1 staining was also observed on the cell surface thereby avoiding false positives due to antibody adsorption to the nanomineral particles as previously described^6^ (see Methods and Supplementary Figure 2 for further details). In Crohn’s disease samples, only occasional weak expression of PD-L1 was observed (n = 177 cells studied; 6 independent patients). In all control tissues, PD-L1 was highly expressed on these same nanomineral positive cells (n = 191 cells studied; 9 independent patients) (p < 0.0004; Figs 2D and 3 and Table 1). The presence of PD-L1 expression on nanomineral positive cells from the ileum of patients with ulcerative colitis (Fig. 3 and Table 1) indicates that the aberrant phenotype is not a generalised phenomenon in inflammatory bowel conditions, but is specific to Crohn’s disease. This also implies that the NAP-pathway specific reduction in PD-L1 expression is not likely to be therapy-induced as both Crohn’s disease and ulcerative colitis patients had been receiving immunomodulatory medications. This is consistent with earlier studies showing that the steroid prednisone does not affect PD-L1 expression on plasmacytoid dendritic cells^22^ and that the suppression we observe is not seen across all cell types.

### NOD2 polymorphisms are not the direct cause of defective PD-L1 expression in Crohn’s disease

Crohn’s disease is associated with polymorphisms in the *NOD2* gene. The NOD2 protein is responsible for cytoplasmic detection of the peptidoglycan fragment muramyl dipeptide, and mice lacking *Nod1* and *Nod2* appear to be defective in PD-L1 expression on APCs involved in the NAP pathway^6^. To check if NOD2 dysfunction alone could explain the defective PD-L1 expression in our Crohn’s disease samples we isolated DNA from Crohn’s disease and control tissue samples and amplified sections of the *NOD2* gene by PCR. These were sequenced to determine the presence, or absence, of the three most common Crohn’s disease-associated NOD2 polymorphisms, namely R702W, G908R and L1007fsincC. Of our six Crohn’s disease tissue samples two were heterozygous for G908R, one was heterozygous for L1007fsincC, one was heterozygous for both R702W and G908R, and two possessed none of the polymorphisms. Interestingly, one of the ulcerative colitis control samples was also heterozygous for L1007fsincC (Table 1). The diversity in polymorphism possession, the presence of L1007fsincC in one of the ulcerative colitis controls, and the lack of polymorphisms in two of the Crohn’s disease subjects, clearly supports that the aberrant expression of PD-L1 is not dictated directly by *NOD2* genotype.

**Figure 4:**
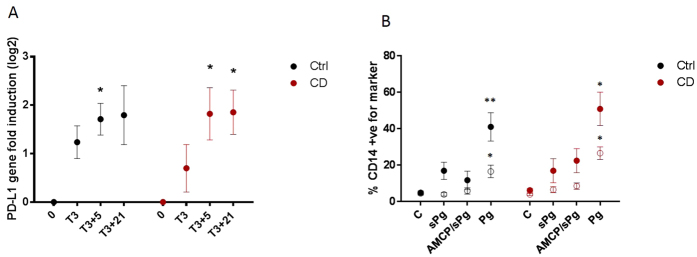
NAP induced PD-L1 expression in peripheral blood mononuclear cells of patients with Crohn’s disease is conserved. (**A**) Time-course of *CD274* (encoding PD-L1) gene expression following an initial 3 hour exposure to particulate Pg (AMCP/sPG) in peripheral blood mononuclear cells from healthy (black; n = 4) and Crohn’s disease (red; n = 6) individuals. Timepoints represent the following: 0 = no stimulation; T3 = expression at end of 3 hour stimulation; T3 + 5 and T3 + 21; expression following, respectively, a further 5 hours and 21 hours incubation in the control medium (i.e. absence of further stimulus). (**B**) PD-L1 (closed symbols) and PD-L2 (open symbols) surface expression following exposure to soluble Pg (sPg), particulate Pg (AMCP/sPg) and crude Pg (Pg) in CD14^+^ cells from healthy (black; n = 4) and Crohn’s disease (red; n = 6) donors. Data was obtained following a 3 hr stimulation and a further 21 hr incubation in control medium and is expressed as Mean ± SEM. Statistical significance of gene induction was calculated by repeated measurement one-way ANOVA with Dunnett’s multiple comparisons; and for protein expression levels using paired two-tailed t-tests. *p < 0.05, **p < 0.01. Ctrl = Controls. CD = Crohn’s disease.

We further examined whether *NOD2* genotype influences the induction of PD-L1 in peripheral cells when stimulated with either free peptidoglycan, or peptidoglycan contained in nanomineral particles (i.e nanoparticles of amorphous magnesium substituted calcium phosphate; AMCP). PBMCs from a new cohort of Crohn’s disease patients, heterozygous for *NOD2* polymorphisms, and from healthy controls, responded in the same manner at both the gene and surface protein levels following stimulation (Fig. 4). In both populations stimulation of PBMCs with peptidoglycan-containing AMCP for 3 hours resulted in weak but significant upregulation of the *CD274* gene after a further 5 hours incubation in control medium (Fig. 4A). Weak surface expression of PD-L1, but not PD-L2, was induced following cell stimulation with both soluble and particulate peptidoglycan (Fig. 4B). However, when using crude preparations of peptidoglycan from *S. Aureus* the levels of surface PD-L1 and PD-L2 increased significantly in both control and Crohn’s disease cohorts (Fig. 4B). These observations fit with our previous work showing that PBMCs from Crohn’s disease patients homozygous for *NOD2* L1007fsincC do not induce PD-L1 upon exposure to muramyl dipeptide, but they do so when stimulated with crude peptidoglycan in a manner presumed to be mediated by a receptor other than NOD2. In contrast, PBMCs from heterozygotes still responded to MDP by increasing PD-L1 expression^7^. Together these findings support our observations in intestinal tissue that the lack of PD-L1 expression in the NAP-pathway of Crohn’s disease patients is a cell (or site) specific phenomenon. This phenotype is consistent with that seen in murine cells defective in peptidoglycan processing^6^ and suggests that an inability to correctly ‘see’ peptidoglycan in Crohn’s disease results in aberrant expression of PD-L1 in APCs stimulated via the NAP pathway.

Collectively, our findings indicate that the APCs of the recently described NAP pathway of intestinal antigen translocation display a selective failure in expression of PD-L1 in Crohn’s disease patients. Consequently these APCs could present the NAP-conjugate derived exogenous antigen, whether in the form of bacterial components or other luminal derived antigen, without concomitant negative co-stimulatory signalling. PD-L1 is involved in the development of regulatory T cells and is sometimes up-regulated during inflammation to restrain tissue damage^8^. Indeed, PD-L1 expression prevented severe CD8^+^ T cell mediated autoimmune enteritis in the iFABP-tOVA transgenic mouse model^13^. Our findings would explain not only the loss of tolerance to the luminal stream that is so commonly considered a hallmark of Crohn’s disease^23^, but also the observation that the earliest visible sign of Crohn’s disease manifests as microscopic ulceration that extends from the sub-epithelial dome of lymphoid patches^20^,^24^. The importance of PD-L1 in controlling intestinal inflammation^13^,^25^ and the normal pattern of PD-L1 expression observed in ulcerative colitis ileum provides clear support for critical involvement of APCs from the NAP pathway in the maintenance of intestinal tolerance. The defective presentation of PD-L1 on NAP pathway APCs in Crohn’s disease may provide the missing link in the Crohn’s disease ‘road map’. By failing to upregulate PD-L1, APCs of the gut lymphoid tissue, which sample luminal peptidoglycan and antigen via the NAP pathway would present antigen in the absence of negative co-stimulation. Failure in PD-L1/PD-1 signalling combined with pro-inflammatory cytokine skewing leads to a gradual and general loss in tolerance towards antigen^26^,^27^,^28^. In this case, infection, or some other ‘adjuvant’ effect, could spark a local immuno-inflammatory response leading to the earliest disease signs of aphthoid ulceration of lymphoid aggregates and follicles. Subsequently, signalling that might normally limit inflammation could cascade. For example, TGF-β in the intestinal environment normally controls regulatory T cell development, however, in the presence of IL-1β, IL-6, IL-21 and IL-23 the emergence and maintenance of Th17 cells occurs^29^ and continuing inflammation may ensue. Restoring PD-L1 signalling in the NAP pathway would therefore present an interesting therapeutic option for the treatment of Crohn’s disease.

## Materials and Methods

### Analysis of intestinal CD274 gene and PD-L1 protein expression from public datasets

PD-L1 is expressed from the *CD274* gene (Gene ID 29126; RefSeq NM_014143). Potentially relevant studies for analysis were identified from the PubMed Database (access on 11/11/2013) using the search term: (whole genome OR transcriptom* OR gene array OR microarray OR PD-L1 OR CD274 OR B7-H1 OR PDCD1L1) AND (crohn* OR colitis OR ibd) AND (gut OR intestin* OR lamina propria OR peyer’s patch OR colon* OR ileum OR ileal OR bowel). Forty five studies (Supplementary Table 1) from 434 hits met our pre-defined inclusion criteria: (a) published in English; (b) data on Crohn’s disease and controls; (c) intestine-derived samples from human subjects; (d) data on *CD274* gene or PD-L1 protein expression, or global gene expression; and (e) original research. Two studies reported *CD274* specific gene and/or PD-L1 protein data (Supplementary Table 2), while the rest provided whole genome expression data. Whole genome expression studies were excluded from further analysis if the data was not publicly accessible or did not contain *CD274* expression data. Ultimately 22 publications from 11 unique datasets deposited on Gene Expression Omnibus (http://www.ncbi.nlm.nih.gov/geo/) or ArrayExpress (http://www.ebi.ac.uk/arrayexpress/) were analysed further (Supplementary Table 3).

Where available, *CD274* expression data was extracted using the web tool GEO2R, offered on Gene Expression Omnibus. GEO2R analyses yielded log_2_ fold changes (FC) which were manually converted into linear FC.


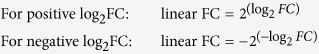


GEO2R analysis further returned the p value adjusted for multiple testing using Benjamini & Hochberg false discovery rate. If GEO2R analysis was not available, pre-processed raw data were accessed on GEO or downloaded from ArrayExpress, and manual calculations performed in Microsoft Excel 2010. Where necessary linear expression values were first log_2_ transformed. The log_2_ average expression value for each condition was used to calculate the fold change:


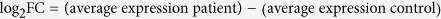


Log_2_ FC was then converted into linear FC as described above. The p value was calculated using a two-tailed student t-test, assuming equal variances.

### Tissue Sections

Fresh anonymised lymphoid patch-containing human ileal tissue specimens used in this study were purchased from a commercial tissue bank (Tissue Solutions, UK) and held for use under HTA licence agreement 12383. Control samples were from the resection margins of patients with intestinal tumours (7 samples) or ulcerative colitis (2 samples), and Crohn’s disease samples (6 samples) were from patients with different disease anatomical locations (Supplementary Table 4). Following collection specimens were snap frozen and embedded in Optimal Cutting Temperature compound. Tissue sections were subsequently cryo-sectioned at 14 μm thickness, collected on SuperFrost® slides (Thermo Scientific, USA) and allowed to air dry for 1 hour at room temperature.

### Immunohistochemistry

Immunohistochemical staining was undertaken in pairs (or multiples thereof) so that one Crohn’s disease section was always accompanied by a control section with identical staining. Prior to staining tissue sections were briefly fixed in 4% formaldehyde (Sigma-Aldrich, UK) for 10 to 15 min to prevent sample degradation. Tissue sections were then blocked using 10% v/v goat serum, 1% w/v BSA, and 0.3 M Glycine. Sections were incubated with primary antibody [mouse anti-human PD-L1 (clone M1H1, eBioscience, USA); mouse anti-human glycoprotein 2 (MBL, USA); and mouse anti-peptidoglycan (clone 3F6B3, AbD Serotec, UK)], followed by incubation with secondary antibody (Alexa Fluor^®^ 568 Goat Anti-mouse IgG [H + L], Invitrogen, UK) and stained for endogenous nanomineral using 0.01 M calcein (Sigma-Aldrich, UK). Nuclei were counterstained with ToPro-3 iodide (Invitrogen, UK) or Hoescht 33342 (Invitrogen, UK). Sections were mounted using ProLong® Gold Antifade Mountant (Invitrogen, UK).

### Confocal microscopy

Confocal imaging was undertaken, at room temperature, in pairs (or multiples thereof) so that one Crohn’s disease section was always accompanied by a control section, and identical laser intensities were used. Detector gains for Crohn’s disease sections were set equal to, or higher than, that of the matched control ensuring that even faint PD-L1 staining above background could be detected for the former. Sections were imaged with a Leica DMIRE2 microscope (Leica Microsystems, Germany) at 488, 568 or 633 nm, fitted with diode Ar/ArKr and HeNe lasers, using either a x20, 0.7 NA multi-immersion lens or a x63, 1.2 NA water objective lens. Data were recorded using the Leica Confocal Software (v2.61) and images processed using ImageJ^30^. Data were collected as 8-bit greyscale images and subsequently assigned appropriate colours. Nuclei are shown grey to enable better visualisation of non-nuclear stains. Identical imaging and data collection routines were applied to Crohn’s disease and control sections. Three-dimensional representations were deconvoluted and reconstructed from z-stacks using Huygens Professional deconvolution software (SVI, The Netherlands).

### Fluorescence Microscopy

Fluorescence imaging was undertaken, at room temperature using a Leica DMI6000B microscope with a x63, 0.7 NA water objective lens and images were captured using a Leica DFC300FX camera. Data were recorded using the Leica Application Suite (v2.1.0R1) and images processed using ImageJ^30^. Data were collected as 8-bit greyscale images and subsequently assigned appropriate colours.

### Determination of cell PD-L1 status

Overall PD-L1 expression in each lymphoid patch was determined by selecting the entire patch at 20x magnification and using ImageJ^30^ to quantify the mean fluorescence intensity in the red channel. PD-L1 status of calcein positive cells was examined on 63x micrographs. All cells showing clear calcein staining were selected, enlarged and their PD-L1 status determined; 3D stacks were used to assist determination in ambiguous cells (Supplementary Figure 2). As antibodies used for staining may adsorb to the nanomineral complexes^6^ cells that showed only overlapping signals from the intracellular calcein stain and the PD-L1 antibody were not considered PD-L1 positive. To be classed as PD-L1 positive clear evidence of separate PD-L1 staining was required (Supplementary Figure 2). Statistical differences were assessed with Mann-Whitney test using GraphPad Prism 6.03.

### NOD2 Genotyping

DNA was extracted from tissue samples using Invitrogen’s PureLink Genomic DNA Mini Kit as per the manufacturer’s instructions. DNA concentration was measured using a NanoDrop spectrophotometer and fragments of the *NOD2* gene amplified by PCR using an Abi 7500 Fast Real Time PCR instrument under the following conditions: 95 °C for 10 min, then 40–55 cycles of 95 °C for 15 seconds and 60 °C for 1 min. The primers used were based on either published primer sets (G908R^31^) or custom designed primers as follows: R702Wfor+M13 5-GTAAAACGACGGCCAGTTGCCCACGATGTGCATCC-3; R702Wrev+M13 5-CAGGAAACAGCTATGACCGATGGAGTGGAAGTGCTTGC-3; L1007fsincC for 5-CCTGCAGTCTCTTTAACTGG-3; and L1007fsincCrev 5-CTTACCAGACTTCCAGGATG-3. PCR amplicons were purified using Qiagen’s MinElute PCR Purification Kit and sequenced at the Department of Biochemistry, University of Cambridge.

### Gene and protein expression in peripheral blood mononuclear cells

Peripheral blood mononuclear cells were isolated by density gradient centrifugation from healthy volunteers (n = 4) or Crohn’s disease patients heterozygotic for *NOD2* polymorphisms (n = 6; R702W – 2 patients; G908R – 1 patient; L1007fsincC – 3 patients). Cells were rested overnight prior to experimentation. Cell suspensions at 1 × 10^6^ cells/ml were incubated at 37 °C in 5% CO_2_ in RPMI 1640 supplemented with 10% fetal calf serum, 2 mM L-glutamine, penicillin (100 U/ml) and streptomycin (100 μg/ml). Cells were stimulated with either 5 μg/mL soluble *E. Coli* peptidoglycan (sPg; Invivogen), 5 μg/mL crude Pg from S.Aureus (Sigma-Aldrich), particulate Pg (AMCP/sPg – soluble Pg incorporated into AMCP particles), or culture media alone. After 3 hours cells were washed in HBSS (Sigma) and re-suspended in fresh culture media for a further 5 or 21 hours. Following incubation, cells were prepared for RNA extraction and *CD274* gene expression analyses as previously described^7^. In parallel experiments, cells were also collected at 3 + 21 hours for analyses of PD-L1 and PD-L2 expression by flow cytometry. Cell pellets were stained with PD-L1 FITC (BD 558065) PD-L2 PE (BD 558066) and CD14 VioBlue (Miltenyi Biotec 130-094-364), according to manufacturers’ instructions. Cells were washed and re-suspended in PBS containing 1% PFA and placed on ice in the dark until acquisition. Single stain and unstained compensation tubes were prepared to compensate for spectral overlap. Cells were acquired on a Cyan-ADP flow cytometer using Summit software for acquisition and analysis, acquiring a minimum of 450,000 events per sample.

### Statistics

Statistical significance of gene induction was calculated by repeated measurement one-way ANOVA with Dunnett’s multiple comparisons; and for protein expression levels using paired two-tailed t-tests. Significance was taken as p < 0.05.

### Ethics Statement

The study and experimental protocols were approved by the Cambridge National Research Ethics Committees (reference 03/296 and 05/Q0108/355) and carried out in accordance with approved guidelines. Peripheral blood mononuclear cells were isolated from the blood of recruited healthy volunteers or Crohn’s disease patients following written informed consent while, for the immunohistochemistry work, tissue sections were purchased anonymised from Tissue Solutions (a commercial tissue bank for which donors provide written informed consent).

## Additional Information

**How to cite this article**: Robertson, J. *et al*. Intestinal APCs of the endogenous nanomineral pathway fail to express PD-L1 in Crohn’s disease. *Sci. Rep*. **6**, 26747; doi: 10.1038/srep26747 (2016).

## Supplementary Material

Supplementary Information

## Figures and Tables

**Table 1:** Nanomineral (calcein) positive and PD-L1 positive cell counts.

Diagnosis	Total number of calcein^+^ cells	Number of calcein^+^ PD-L1^+^ cells (% in brackets)	NOD2 Genotype
Crohn’s disease	44	5 (11.4)	Wild-type
	19	0 (0)	L1007fsincC
	18	2 (11.1)	G908R
	5	0 (0)	Wild-type
	53	3 (5.7)	R702W, G908R
	38	2 (5.3)	G908R
Carcinoma (controls)	17	14 (82.4)	Wild-type
	23	21 (91.3)	Wild-type
	7	7 (100)	Wild-type
	59	52 (88.1)	Wild-type
	17	17 (100)	Wild-type
	16	15 (93.8)	Wild-type
	10	10 (100)	Wild-type
Ulcerative colitis (controls)	16	15 (93.75)	Wild-type
	26	26 (100)	L1007fsincC
