# Supplementary material for: Intestinal APCs of the endogenous nanomineral pathway fail to express PD-L1 in Crohn’s disease
Source: Sci Rep. 2016 May 26;6:26747. doi: 10.1038/srep26747 (PMC4880906; doi:10.1038/srep26747)
Supplement: Supplementary Information [file srep26747-s1.pdf]

Supplementary Information

Intestinal APCs of the endogenous nanomineral pathway fail to express PD-L1 in Crohn's disease

Jack Robertson[1], Carolin T Haas[1], Laetitia C Pele[1], Tom P Monie[1], Charles Charalambos[1], Miles Parkes[2], Rachel E Hewitt[1] and Jonathan J Powell[1*].

[1] Medical Research Council Human Nutrition Research, Elsie Widdowson Laboratory, 120 Fulbourn Road, Cambridge CB1 9NL, UK.

[2]Department of Medicine, University of Cambridge School of Clinical Medicine, Cambridge Biomedical Campus, Cambridge, United Kingdom

[*] Corresponding author: Professor Jonathan Powell: Jonathan.Powell@mrc-hnr.cam.ac.uk

Supplementary Figure 1:

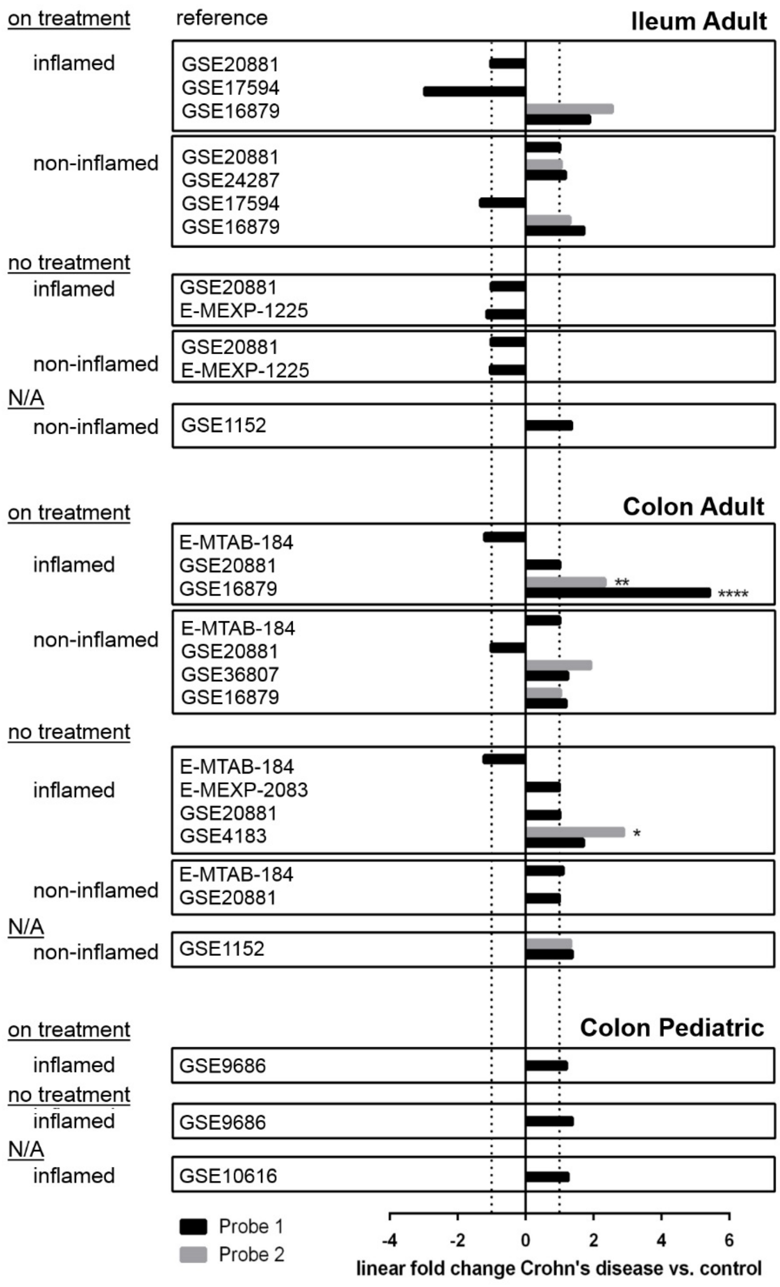

Figure S1: Schematic summary of intestinal *CD274* gene expression comparing Crohn's disease patients and controls. Dotted lines represent linear fold change of -1 and +1. Only asterisked data reached statistical significance (* p = 0.01, ** p = 0.006 and *** p = 0.0001). Detailed information on sample grouping, probe IDs and references can be found in Supplementary Table 1. All data obtained from publically available whole genome expression studies.

Supplementary Figure 2:

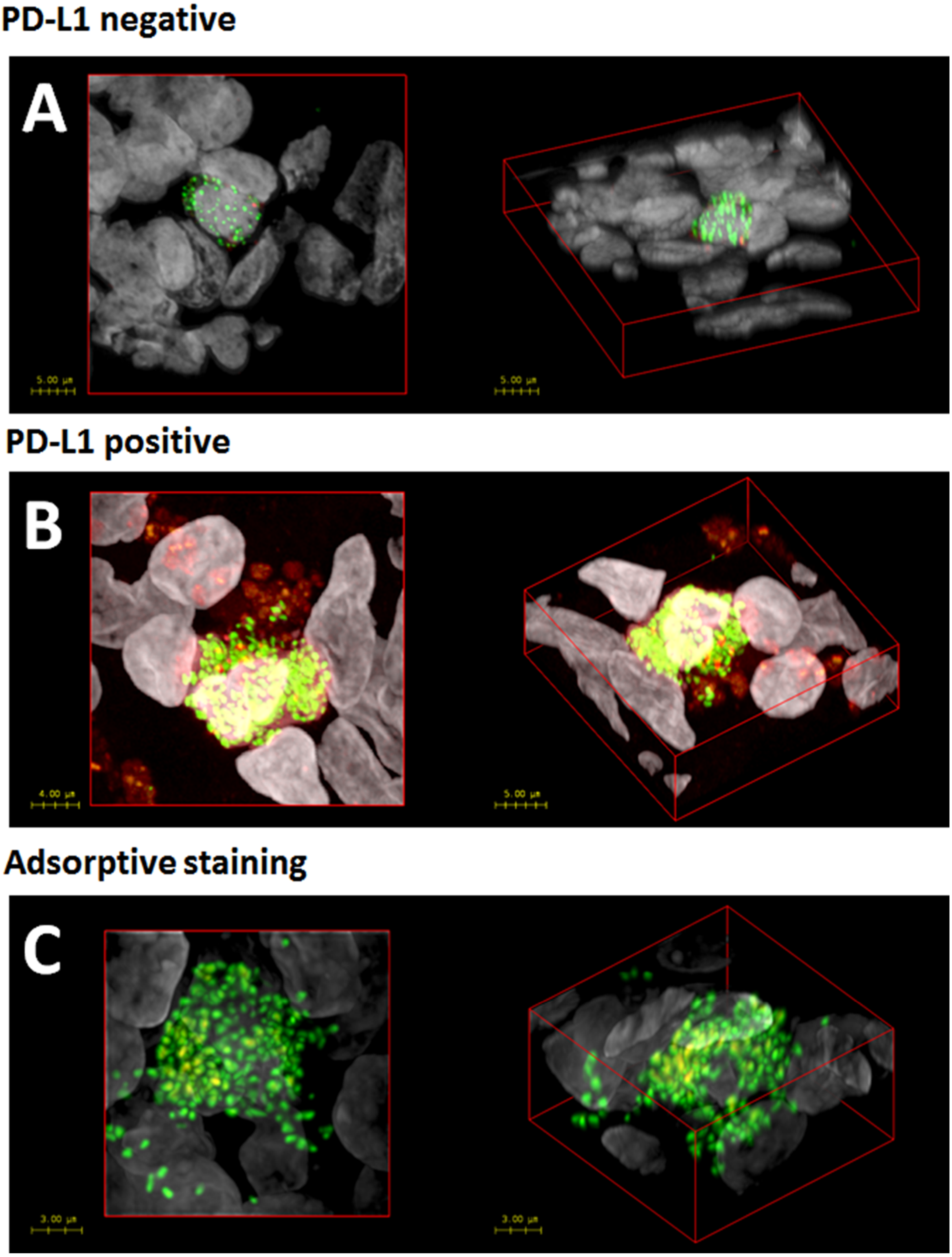

Figure S2: Representative 3D stacks of nanomineral positive cells detailing different patterns of PD-L1 staining. Deconvoluted reconstructions of z-stacks, giving three dimensional representations of PD-L1 staining on nanomineral positive (calcein stained; green)

cells showing (**A**) no concomitant cellular expression of PD-L1, (**B**) PD-L1 expression (red), and (**C**) adsorptive-only interactions (orange/yellow) between the PD-L1 antibody and the nanomineral.

Supplementary Table 1

Studies containing PD-L1 specific or whole genome expression data from Crohn's disease *versus* control intestine

No.	Reference	Inclusion / Exclusion[a]
1	Montero-Meléndez T, Llor X, García-Planella E, Perretti M, Suárez A. Identification of novel predictor classifiers for inflammatory bowel disease by gene expression profiling. PLoS One. 2013 Oct 14;8(10):e76235. doi: 10.1371/journal.pone.0076235. PubMed PMID: 24155895; PubMed Central PMCID: PMC3796518.	Included GSE36807
2	Østvik AE, Granlund AV, Torp SH, Flatberg A, Beisvåg V, Waldum HL, Flo TH, Espevik T, Damås JK, Sandvik AK. Expression of Toll-like receptor-3 is enhanced in active inflammatory bowel disease and mediates the excessive release of lipocalin 2. Clin Exp Immunol. 2013 Sep;173(3):502-11. doi: 10.1111/cei.12136. PubMed PMID: 23668802.	Included E-MTAB-184
3	Walker SJ, Fortunato J, Gonzalez LG, Krigsman A. Identification of unique gene expression profile in children with regressive autism spectrum disorder (ASD) and ileocolitis. PLoS One. 2013;8(3):e58058. doi: 10.1371/journal.pone.0058058. Epub 2013 Mar 8. PubMed PMID: 23520485; PubMed Central PMCID: PMC3592909.	Excluded Data set not available
4	Granlund Av, Flatberg A, Østvik AE, Drozdov I, Gustafsson BI, Kidd M, Beisvag V, Torp SH, Waldum HL, Martinsen TC, Damås JK, Espevik T, Sandvik AK. Whole genome gene expression meta-analysis of inflammatory bowel disease colon mucosa demonstrates lack of major differences between Crohn's disease and ulcerative colitis. PLoS One. 2013;8(2):e56818. doi: 10.1371/journal.pone.0056818. Epub 2013 Feb 13. PubMed PMID: 23468882; PubMed Central PMCID: PMC3572080.	Included E-MTAB-184
5	Zhang T, DeSimone RA, Jiao X, Rohlf FJ, Zhu W, Gong QQ, Hunt SR, Dassopoulos T, Newberry RD, Sodergren E, Weinstock G, Robertson CE, Frank DN, Li E. Host genes related to paneth cells and xenobiotic metabolism are associated with shifts in human ileum-associated microbial composition. PLoS One. 2012;7(6):e30044. doi: 10.1371/journal.pone.0030044. Epub 2012 Jun 13. PubMed PMID: 22719822; PubMed Central PMCID: PMC3374611.	Included GSE24287
6	Hetzenecker AM, Seidl MC, Kosovac K, Herfarth H, Kellermeier S, Obermeier F, Falk W, Schoelmerich J, Hausmann M, Rogler G. Downregulation of the ubiquitin-proteasome system in normal colonic macrophages and reinduction in inflammatory bowel disease. Digestion. 2012;86(1):34-47. doi: 10.1159/000336353. Epub 2012 Jun 15. PubMed PMID: 22710419.	Excluded Data set not available
7	Ostvik AE, Granlund AV, Bugge M, Nilsen NJ, Torp SH, Waldum HL, Damås JK, Espevik T, Sandvik AK. Enhanced expression of CXCL10 in inflammatory bowel disease: potential role of mucosal Toll-like receptor 3 stimulation. Inflamm Bowel Dis. 2013 Feb;19(2):265-74. doi: 10.1002/ibd.23034. PubMed PMID: 22685032.	Included E-MTAB-184
8	Zhang T, Song B, Zhu W, Xu X, Gong QQ, Morando C, Dassopoulos T, Newberry RD,<br>Hunt SR, Li E. An ileal Crohn's disease gene signature based on whole human genome expression profiles of disease unaffected ileal mucosal biopsies. PLoS One. 2012;7(5):e37139. doi: 10.1371/journal.pone.0037139. Epub 2012 May 14. PubMed PMID: 22606341; PubMed Central PMCID: PMC3351422.	Included GSE24287
9	Ohtsuka Y, Jimbo K, Inage E, Mori M, Yamakawa Y, Aoyagi Y, Suzuki M, Kudo T, Suzuki R, Shimizu T. Microarray analysis of mucosal biopsy specimens in neonates with rectal bleeding: is it really an allergic disease? J Allergy Clin Immunol. 2012 Jun;129(6):1676-8. doi: 10.1016/j.jaci.2012.01.042. Epub 2012 Feb 11. PubMed PMID: 22326485.	Excluded Data set not available
10	LaPointe LC, Pedersen SK, Dunne R, Brown GS, Pimlott L, Gaur S, McEvoy A, Thomas M, Wattchow D, Molloy PL, Young GP. Discovery and validation of molecular	Excluded

	biomarkers for colorectal adenomas and cancer with application to blood testing. PLoS One. 2012;7(1):e29059. doi: 10.1371/journal.pone.0029059. Epub 2012 Jan 19. PubMed PMID: 22276102; PubMed Central PMCID: PMC3261845.	Data set for CD not available
11	Granlund Av, Beisvag V, Torp SH, Flatberg A, Kleveland PM, Ostvik AE, Waldum HL, Sandvik AK. Activation of REG family proteins in colitis. Scand J Gastroenterol. 2011 Nov;46(11):1316-23. doi: 10.3109/00365521.2011.605463. PubMed PMID: 21992413; PubMed Central PMCID: PMC3212911.	Included E-MTAB-184
12	Funke B, Lasitschka F, Roth W, Penzel R, Meuer S, Saile M, Gretz N, Sido B, Schirmacher P, Autschbach F. Selective downregulation of retinoic acid-inducible gene I within the intestinal epithelial compartment in Crohn's disease. Inflamm Bowel Dis. 2011 Sep;17(9):1943-54. doi: 10.1002/ibd.21572. Epub 2011 Jan 6. PubMed PMID: 21830273.	Included E-MEXP-2083 and E-MEXP-1225
13	Nimmo ER, Stevens C, Phillips AM, Smith A, Drummond HE, Noble CL, Quail M, Davies G, Aldhous MC, Wilson DC, Satsangi J. TLE1 modifies the effects of NOD2 in the pathogenesis of Crohn's disease. Gastroenterology. 2011 Sep;141(3):972-981.e1-2. doi: 10.1053/j.gastro.2011.05.043. Epub 2011 May 27. PubMed PMID: 21699783.	Included GSE20881
14	Arijs I, De Hertogh G, Machiels K, Van Steen K, Lemaire K, Schraenen A, Van Lommel L, Quintens R, Van Assche G, Vermeire S, Schuit F, Rutgeerts P. Mucosal gene expression of cell adhesion molecules, chemokines, and chemokine receptors in patients with inflammatory bowel disease before and after infliximab treatment. Am J Gastroenterol. 2011 Apr;106(4):748-61. doi: 10.1038/ajg.2011.27. Epub 2011 Feb 15. PubMed PMID: 21326222.	Included GSE16879
15	Arijs I, Quintens R, Van Lommel L, Van Steen K, De Hertogh G, Lemaire K, Schraenen A, Perrier C, Van Assche G, Vermeire S, Geboes K, Schuit F, Rutgeerts P. Predictive value of epithelial gene expression profiles for response to infliximab in Crohn's disease. Inflamm Bowel Dis. 2010 Dec;16(12):2090-8. doi: 10.1002/ibd.21301. PubMed PMID: 20848504.	Included GSE16879
16	Noble CL, Abbas AR, Lees CW, Cornelius J, Toy K, Modrusan Z, Clark HF, Arnott ID, Penman ID, Satsangi J, Diehl L. Characterization of intestinal gene expression profiles in Crohn's disease by genome-wide microarray analysis. Inflamm Bowel Dis. 2010 Oct;16(10):1717-28. doi: 10.1002/ibd.21263. PubMed PMID: 20848455.	Included GSE20881
17	Vermeulen N, Vermeire S, Arijs I, Michiels G, Ballet V, Derua R, Waelkens E, Van Lommel L, Schuit F, Rutgeerts P, Bossuyt X. Seroreactivity against glycolytic enzymes in inflammatory bowel disease. Inflamm Bowel Dis. 2011 Feb;17(2):557-64. doi: 10.1002/ibd.21388. PubMed PMID: 20629101.	Excluded Data set not available
18	Slavova N, Drescher A, Visekruna A, Dullat S, Kroesen AJ, Ritz JP, Buhr HJ. NALP expression in Paneth cells provides a novel track in IBD signaling. Langenbecks Arch Surg. 2010 Apr;395(4):351-7. doi: 10.1007/s00423-010-0611-8. Epub 2010 Mar 24. PubMed PMID: 20333398.	Excluded Data set not available
19	Hamm CM, Reimers MA, McCullough CK, Gorbe EB, Lu J, Gu CC, Li E, Dieckgraefe BK, Gong Q, Stappenbeck TS, Stone CD, Dietz DW, Hunt SR. NOD2 status and human ileal gene expression. Inflamm Bowel Dis. 2010 Oct;16(10):1649-57. doi: 10.1002/ibd.21208. PubMed PMID: 20155851.	Included GSE17594
20	Arijs I, De Hertogh G, Lemaire K, Quintens R, Van Lommel L, Van Steen K, Leemans P, Cleynen I, Van Assche G, Vermeire S, Geboes K, Schuit F, Rutgeerts P. Mucosal gene expression of antimicrobial peptides in inflammatory bowel disease before and after first infliximab treatment. PLoS One. 2009 Nov 24;4(11):e7984. doi: 10.1371/journal.pone.0007984. PubMed PMID: 19956723; PubMed Central PMCID: PMC2776509.	Included GSE16879
21	Nielsen OH, Bjerrum JT, Csillag C, Nielsen FC, Olsen J. Influence of smoking on colonic gene expression profile in Crohn's disease. PLoS One. 2009 Jul	Excluded E-TABM-118, no

	15;4(7):e6210. doi: 10.1371/journal.pone.0006210. PubMed PMID: 19603079; PubMed Central PMCID: PMC2708910.	processed data available
22	Olsen J, Gerds TA, Seidelin JB, Csillag C, Bjerrum JT, Troelsen JT, Nielsen OH. Diagnosis of ulcerative colitis before onset of inflammation by multivariate modeling of genome-wide gene expression data. Inflamm Bowel Dis. 2009 Jul;15(7):1032-8. doi: 10.1002/ibd.20879. PubMed PMID: 19177426.	Excluded E-TABM-118, no processed data available
23	Lang M, Schlechtweg M, Kellermeier S, Brenmoehl J, Falk W, Schölmerich J, Herfarth H, Rogler G, Hausmann M. Gene expression profiles of mucosal fibroblasts from strictured and nonstrictured areas of patients with Crohn's disease. Inflamm Bowel Dis. 2009 Feb;15(2):212-23. doi: 10.1002/ibd.20735. PubMed PMID: 18839425.	Excluded Data set not available
24	Galamb O, Györffy B, Sipos F, Spisák S, Németh AM, Miheller P, Tulassay Z, Dinya E, Molnár B. Inflammation, adenoma and cancer: objective classification of colon biopsy specimens with gene expression signature. Dis Markers. 2008;25(1):1-16. PubMed PMID: 18776587.	Included GSE4183
25	Srivastava MD, Kulaylat MN. Gene expression profiles of late colonic Crohn's disease. J Med. 2004;35(1-6):233-55. PubMed PMID: 18084881.	Excluded Data set not available
26	Carey R, Jurickova I, Ballard E, Bonkowski E, Han X, Xu H, Denson LA. Activation of an IL-6:STAT3-dependent transcriptome in pediatric-onset inflammatory bowel disease. Inflamm Bowel Dis. 2008 Apr;14(4):446-57. PubMed PMID: 18069684; PubMed Central PMCID: PMC2581837.	Included GSE9686
27	Orsó E, Moehle C, Boettcher A, Szakszon K, Werner T, Langmann T, Liebisch G, Buechler C, Ritter M, Kronenberg F, Dieplinger H, Bornstein SR, Stremmel W, Schmitz G. The satiety factor apolipoprotein A-IV modulates intestinal epithelial permeability through its interaction with alpha-catenin: implications for inflammatory bowel diseases. Horm Metab Res. 2007 Aug;39(8):601-11. PubMed PMID: 17712726.	Included GSE1152
28	Csillag C, Borup R, Olsen J, Nielsen FC, Nielsen OH. Treatment response and colonic gene expression in patients with Crohn's disease. Scand J Gastroenterol. 2007 Jul;42(7):834-40. PubMed PMID: 17558907.	Excluded E-TABM-118, no processed data available
29	Zahn A, Moehle C, Langmann T, Ehehalt R, Autschbach F, Stremmel W, Schmitz G. Aquaporin-8 expression is reduced in ileum and induced in colon of patients with ulcerative colitis. World J Gastroenterol. 2007 Mar 21;13(11):1687-95. PubMed PMID: 17461471.	Included GSE1152
30	Csillag C, Nielsen OH, Vainer B, Olsen J, Dieckgraefe BK, Hendel J, Vind I, Dupuy C, Nielsen FC, Borup R. Expression of the genes dual oxidase 2, lipocalin 2 and regenerating islet-derived 1 alpha in Crohn's disease. Scand J Gastroenterol. 2007 Apr;42(4):454-63. PubMed PMID: 17454855.	Excluded E-TABM-118, no processed data available
31	Csillag C, Nielsen OH, Borup R, Olsen J, Bjerrum JT, Nielsen FC. CARD15 status and familial predisposition for Crohn's disease and colonic gene expression. Dig Dis Sci. 2007 Aug;52(8):1783-9. Epub 2007 Apr 5. PubMed PMID: 17410442.	Excluded E-TABM-118, no processed data available
32	Wu F, Dassopoulos T, Cope L, Maitra A, Brant SR, Harris ML, Bayless TM, Parmigiani G, Chakravarti S. Genome-wide gene expression differences in Crohn's disease and ulcerative colitis from endoscopic pinch biopsies: insights into distinctive pathogenesis. Inflamm Bowel Dis. 2007 Jul;13(7):807-21. PubMed PMID: 17262812.	Excluded GSE6731, does not contain data on PD-L1[b]
33	Galamb O, Sipos F, Dinya E, Spisak S, Tulassay Z, Molnar B. mRNA expression, functional profiling and multivariate classification of colon biopsy specimen by cDNA overall glass microarray. World J Gastroenterol. 2006 Nov 21;12(43):6998-7006. PubMed PMID: 17109495.	Excluded Data set not available
34	Moehle C, Ackermann N, Langmann T, Aslanidis C, Kel A, Kel-Margoulis O,	Included

	Schmitz-Madry A, Zahn A, Stremmel W, Schmitz G. Aberrant intestinal expression and allelic variants of mucin genes associated with inflammatory bowel disease. J Mol Med (Berl). 2006 Dec;84(12):1055-66. Epub 2006 Oct 21. PubMed PMID: 17058067.	GSE1152
35	Csillag C, Nielsen OH, Borup R, Nielsen FC, Olsen J. Clinical phenotype and gene expression profile in Crohn's disease. Am J Physiol Gastrointest Liver Physiol. 2007 Jan;292(1):G298-304. Epub 2006 Sep 7. PubMed PMID: 16959948.	Excluded E-TABM-118, no processed data available
36	Heimerl S, Moehle C, Zahn A, Boettcher A, Stremmel W, Langmann T, Schmitz G. Alterations in intestinal fatty acid metabolism in inflammatory bowel disease. Biochim Biophys Acta. 2006 Mar;1762(3):341-50. Epub 2006 Jan 5. PubMed PMID: 16439103.	Included GSE1152
37	Costello CM, Mah N, Häsler R, Rosenstiel P, Waetzig GH, Hahn A, Lu T, Gurbuz Y, Nikolaus S, Albrecht M, Hampe J, Lucius R, Klöppel G, Eickhoff H, Lehrach H, Lengauer T, Schreiber S. Dissection of the inflammatory bowel disease transcriptome using genome-wide cDNA microarrays. PLoS Med. 2005 Aug;2(8):e199.<br>Epub 2005 Aug 23. PubMed PMID: 16107186; PubMed Central PMCID: PMC1188246.	Excluded GSE1710, does not contain data on PD-L1[b]
38	Langmann T, Moehle C, Mauerer R, Scharl M, Liebisch G, Zahn A, Stremmel W, Schmitz G. Loss of detoxification in inflammatory bowel disease: dysregulation of pregnane X receptor target genes. Gastroenterology. 2004 Jul;127(1):26-40. PubMed PMID: 15236169.	Included GSE1152
39	Nakazawa A, Dotan I, Brimnes J, Allez M, Shao L, Tsushima F, Azuma M, Mayer L. The expression and function of costimulatory molecules B7H and B7-H1 on colonic epithelial cells. Gastroenterology. 2004 May;126(5):1347-57. PubMed PMID: 15131796.	Included (PD-L1 PCR and flow cytometry)
40	Dooley TP, Curto EV, Reddy SP, Davis RL, Lambert GW, Wilborn TW, Elson CO. Regulation of gene expression in inflammatory bowel disease and correlation with IBD drugs: screening by DNA microarrays. Inflamm Bowel Dis. 2004 Jan;10(1):1-14. PubMed PMID: 15058520.	Excluded Data set not available
41	Kanai T, Totsuka T, Uraushihara K, Makita S, Nakamura T, Koganei K, Fukushima T, Akiba H, Yagita H, Okumura K, Machida U, Iwai H, Azuma M, Chen L, Watanabe M.<br>Blockade of B7-H1 suppresses the development of chronic intestinal inflammation. J Immunol. 2003 Oct 15;171(8):4156-63. PubMed PMID: 14530338.	Included (PD-L1 IHC and flow cytometry)
42	Uthoff SM, Eichenberger MR, Lewis RK, Fox MP, Hamilton CJ, McAuliffe TL, Grimes HL, Galandiuk S. Identification of candidate genes in ulcerative colitis and Crohn's disease using cDNA array technology. Int J Oncol. 2001 Oct;19(4):803-10. PubMed PMID: 11562759.	Excluded Not retrieved
43	Lawrance IC, Fiocchi C, Chakravarti S. Ulcerative colitis and Crohn's disease: distinctive gene expression profiles and novel susceptibility candidate genes. Hum Mol Genet. 2001 Mar 1;10(5):445-56. PubMed PMID: 11181568.	Excluded Data set not available
44	Dieckgraefe BK, Stenson WF, Korzenik JR, Swanson PE, Harrington CA. Analysis of mucosal gene expression in inflammatory bowel disease by parallel oligonucleotide arrays. Physiol Genomics. 2000 Nov 9;4(1):1-11. PubMed PMID: 11074008.	Excluded Data set not available
45	Kugathasan S, Baldassano RN, Bradfield JP, Sleiman PM et al. Loci on 20q13 and 21q22 are associated with pediatric-onset inflammatory bowel disease. Nat Genet 2008 Oct;40(10):1211-5. PMID: 18758464	Included, GSE10616

[a] See method section for inclusion/exclusion criteria. For whole genome expression studies accession numbers for the data set on Gene Expression Omnibus (starting with GSE) or on ArrayExpress (starting with E-) are shown.

[b] Data set was searched for gene descriptions PD-L1, B7-H1 or programmed cell death 1, gene name CD274, gene ID number 29126, UniGene name Hs.521989, UniGene number 912736, RefSeq NM_014143 and GenBank Accession number AF233516

Supplementary Table 2

Studies investigating intestinal PD-L1 expression at gene and/or protein level. CD = Crohn's disease, IBD = inflammatory bowel disease, UC = ulcerative colitis.

Ref. No. (as listed in Supplementary Table 1)	Sample description	Site	Outcome
41	No age information<br><br>Controls:<br>colon cancer patients<br><br>Patients:<br>Primary Crohn's disease location was ileal (n=6), ileo-colonic (n=6), or colonic (n=4)	Controls:<br>17 surgical colonic and ileal specimens<br><br>Patients:<br>12 surgical specimens and four biopsy specimens comprising inflamed and un-inflamed areas of intestinal mucosa (no further specification)	<u>Immunohistochemistry:</u><br>PD-L1$^+$ mononuclear cells in the lamina propria were markedly increased in inflamed mucosa from CD patients compared with controls<br><br><u>Flow cytometry of isolated lamina propria mononuclear cells (LPMCs):</u><br>CD compared to control: increased CD3$^+$ PD-L1$^+$ LPMCs (p=0.048), no difference in CD19$^+$PD-L1$^+$ LP B cells (p=0.5887), increased macrophage-gated PD-L1$^+$ cells (p=0.021)
39	No age information<br><br>Controls:<br>Cancer patients undergoing bowel resection<br><br>Patients:<br>IBD patients undergoing bowel resection	Surgical specimens from colon (no information on inflamed/non-inflamed areas)<br><br>For controls, samples were taken at least 10 cm away from the tumour	<u>Polymerase chain reaction of isolated colonic epithelial cells:</u><br>PD-L1 mRNA expression in both controls (n=6) and IBD (n=10, no difference between CD and UC)<br><br><u>Flow cytometry of isolated colonic epithelial cells:</u><br>Significantly higher PD-L1 protein expression in CD vs. control (n=5 for both groups, $p < 0.05$)

Supplementary Table 3

Studies that investigate global gene expression in the intestine of Crohn's disease patients *versus* control, and that were used for *CD274* analysis. CD = Crohn's disease, IBD = inflammatory bowel disease, UC = ulcerative colitis, FC = fold change.

	**Accession no. (and ref. no. as listed in Supplementary Table 1)**	**Sample description**	**Site**	**Analysis technical details**	**Samples for *CD274* analysis**	***CD274* analysis details**
1	GSE16879 (ref. 14, 15, 20)	Adult subjects<br><br>Controls:<br>endoscopy for screening for polyps<br><br>Patients:<br>refractory to corticosteroids and/or immunosuppression, and presenting with Crohn's ileitis (CDi) or Crohn's colitis (CDc)<br>→ samples were taken before and after infliximab treatment<br>→ information on anti-inflammatory medication independent of infliximab treatment was given only as summary % but not for individuals. For our *CD274* analysis we assigned all samples to 'on treatment'.	Biopsies from sites of active inflammation (i.e., ileum for CDi and colon for CDc) but at a distance of ulcerations. In the case of healing at control endoscopy, the biopsies were obtained in the areas where lesions were present before therapy.	Affymetrix Human Genome U133 Plus 2.0 Arrays<br><br>Probe ID 1: 223834_at<br><br>Probe ID 2: 227458_at	Control ileum n=6<br>Inflamed CD ileum n=28<br>Non-inflamed CD ileum n=8<br><br>Control colon n=6<br>Inflamed CD colon n=26<br>Non-inflamed CD colon n=11<br><br>All samples taken before infliximab treatment as well as samples from non-responders taken after infliximab treatment were assigned to the group 'inflamed'. The group 'non-inflamed'	GEO2R

					encompasses samples taken after infliximab treatment from responders (colon) or partial responders (ileum) as defined in the original study.	
2	GSE9686 (ref. 26)	Pediatric subjects<br><br>Controls:<br>colonoscopy (no indication given)<br><br>Patients:<br>- at diagnosis<br>- active treated CD	Colon biopsies, in the case of patients taken from an area of active disease in the ascending colon or the most proximal area of active disease if the ascending colon was endoscopically normal	Human Genome HG-U133 Plus 2.0 array from Affymetrix<br><br>Probe ID:<br>NM_014143_at	Control colon n=8<br><br>Inflamed CD colon (no treatment) n=11<br>Inflamed CD colon (on treatment) n=9	GEO2R
3	GSE4183 (ref. 24)	Adult subjects<br><br>Controls:<br>endoscopy (no indication given)<br><br>Patients:<br>active CD	Biopsy samples were taken from the colon during the endoscopical intervention before treatment<br><br>→ no details given as to whether endoscopy was performed prior to a specific treatment only and, therefore, whether patients took any medication at the time of sampling. For our *CD274* analysis we assigned all samples to 'no treatment'.	HGU133 Plus2.0 array<br><br>Probe ID 1:<br>223834_at<br><br>Probe ID 2:<br>227458_at	Control colon n=8<br><br>Inflamed CD colon n=5	GEO2R

4	GSE17594<br>(ref. 19)	Adult subjects<br><br>Controls:<br>undergoing colectomy for colonic neoplasm or colonic inertia<br><br>Patients:<br>Patients were genotyped for NOD2 (Leu1007fsInsC, R702W and G908R).<br>$NOD2^{R}$ patients (R = risk alleles) carry at least 1 of the 3 risk alleles and include $NOD2^{R/R}$ and $NOD2^{R/NR}$.<br>$NOD2^{NR}$ patients carry no risk allele ($NOD2^{NR/NR}$).<br>All patients were on treatment, including anti-inflammatory medication and/or antibiotics.	Biopsies of macroscopically disease-affected and -unaffected areas of ileal mucosa were collected from fresh pathologic specimens	Agilent Whole Human Genome Arrays (Agilent No. G4410A)	Control ileum n=4<br><br>Non-inflamed CD colon n=5<br>Inflamed CD colon n=5<br><br>For our analysis, samples were distinguished only in terms of inflamed/non-inflamed and not by NOD2 genotype as there was no effect of genotype sub-groups on *CD274* outcome (data not shown).	No data for GEO2R available.<br>Raw data (log-transformed) was accessed on GEO and values for *CD274* identified with the Gene ID 29126. No data for PD-L1 available for 6 samples. Manual calculation of FC and p value.
5	GSE10616<br>(ref. 45)	Pediatric subjects<br><br>Controls:<br>no indication for surgery given<br><br>Patients:<br>Presenting with either ileo-colonic or colonic active CD, both at diagnosis or during treatment.<br>→ No information given that allowed sample assignment to groups ‘on treatment’ or ‘no treatment’	Biopsies were taken from affected segments of the ascending colon	Affymetrix GeneChip Human Genome HG-U133 Plus 2.0 array<br><br>Probe ID:<br>NM_014143_at	Control colon n=11<br><br>Inflamed CD colon n=32<br><br>All samples were derived from colon and, for our analysis, were not further distinguished into ileo-colonic and colonic disease as there was no effect of disease sub-type on *CD274* outcome (data not shown).	GEO2R

6	GSE1152<br>(ref. 27, 29, 34, 36, 38)	Adult subjects<br><br>Controls:<br>screening for colon cancer<br><br>Patients:<br>No information given that allowed sample assignment to groups ‘on treatment’ or ‘no treatment’	Biopsies from resected terminal ileum or transverse colon.<br><br>For patients specimens were taken from non-inflamed regions 10 cm distant from pathologic areas. Unaffected areas were defined as mucosa regions without any macroscopic/ endoscopic signs of inflammation.	Affymetrix HGU133A and HGU133B GeneChips<br><br>Samples were hybridized on both arrays. Only HGU133B data contained information on *CD274* and, therefore, was used for our analysis solely.<br>Data set GPL97 which contains all probe sets used in HGU133B gene arrays was downloaded from GEO and Affymetrix Probe Set IDs for *CD274* were identified with the Gene Name CD274.<br><br>Probe ID 1:<br>223834_at<br><br>Probe ID 2:<br>227458_at	Control ileum n=1 (pooled from 4 donors)<br>Control colon n=1 (pooled from 4 donors)<br><br>Non-inflamed CD ileum n=1 (pooled from 4 donors)<br>Non-inflamed CD colon n=1 (pooled from 4 donors)	No data for GEO2R available.<br>Raw data (linear) were accessed on GEO and values for PD-L1 identified with Affymetrix Probe IDs 223834_at and 227458_at. Manual calculation of FC and p value. For control ileum only probe 2 yielded a detectable outcome. No student t test could be performed because each group contained only one data point (pooled from n=4 subjects).

7	GSE36807<br>(ref. 1)	Adult subjects<br><br>Controls:<br>colorectal cancer screening<br><br>Patients:<br>Established CD<br>→ Information on anti-inflammatory medication was given only as summary % but not for individuals. For our *CD274* analysis we assigned all samples to 'on treatment'.	Endoscopic pinch biopsies of macroscopically non-inflamed mucosa were taken from ascending colon	Human Genome U133 plus 2.0 arrays<br><br>Probe ID 1:<br>223834_at<br><br>Probe ID 2:<br>227458_at	Control colon n=7<br><br>Non-inflamed CD colon n=13	GEO2R
8	GSE20881<br>(ref. 13, 16)	Adult subjects<br><br>Controls (n=31):<br>Normal colonoscopies after screening for colon cancer, irritable bowel syndrome or other indications (n=23). Abnormal inflamed colonic biopsies for n=8.<br><br>Patients (n=53):<br>Presenting with active or quiescent disease, distinguished by the Harvey Bradshaw score (≥ 4 for active disease). Patients were classified 'on treatment' if they received anti-inflammatory medication (i.e., Asacol, Pentaza, Azathioprine, Mesalazine, Aza, Sulphasalazine, Salazopyrin, Prednisolone or Mercaptopurine)	Paired biopsies were taken from the terminal ileum and three sites in the colon (sigmoid, ascending, descending) during colonoscopy	Agilent Whole Human Genome Microarrays<br><br>Probe ID:<br>33799	Control ileum n=6<br>Control colon n=67<br><br>On treatment:<br>Inflamed CD ileum n=8<br>Non-inflamed CD ileum n=1<br>Inflamed CD colon n=35<br>Non-inflamed CD colon n=14<br><br>No treatment:<br>Inflamed CD ileum n=6<br>Non-inflamed CD ileum n=2<br>Inflamed CD colon n=24<br>Non-inflamed CD colon n=9	GEO2R

					For our analysis, samples were distinguished for site only by ileum/colon as there was no effect of colonic sub-location on *CD274* outcome (data not shown).	
9	GSE24287 (ref. 5, 8)	Adult subjects<br><br>Controls:<br>undergoing colectomy (for colon cancer, colonic adenomas, colonic inertia, diverticulosis or, in one case, for a foreign body with perforation)<br><br>Patients:<br>Presenting with ileal CD. Patients were genotyped for NOD2 and ATG16L1 and dataset comprises wildtype, homo- and heterozygous genotypes. Genotype information was not given for individual samples. Information on anti-inflammatory medication was given only as summary % but not for individuals. For our *CD274* analysis we assigned all samples to 'on treatment'.	Ex-vivo biopsies from the macroscopically disease unaffected proximal margin of freshly resected pathologic ileum specimens	Agilent Whole Human Genome Arrays (Agilent No. G4412A)<br><br>Probe ID 1: A_23_P338479<br><br>Probe ID 2: A_23_P256487	Control ileum n=25<br><br>Non-inflamed CD ileum n=47	GEO2R

10	E-MEXP-2083 (colon) and E-MEXP-1225 (ileum)<br>(ref. 12)	Adult subjects<br><br>Controls:<br>diagnosed with adenoma/ adenomacarcinoma of the colon or pancreatic cancer<br><br>Patients:<br>Patients in this study were not under anti-inflammatory medication. For 2 out of 3 patients where colon samples were taken information about anti-inflammatory medication other than corticosteroids was not available. For our *CD274* analysis we assigned all samples to 'no treatment'.	Tissue samples from macroscopically inflamed as well as macroscopically non-involved gut regions with subsequent final classification through histopathological examination. Epithelium excision for microarray analysis by laser microdissection.	Affymetrix HGU133 Plus 2.0 GeneChips oligonucleotide arrays<br><br>Array design was downloaded from ArrayExpress and *CD274* probe ID identified with PD-L1 RefSeq NM_014143.<br><br>Probe ID:<br>223834_at	Control colon n=3<br>Control ileum n=3<br><br>Inflamed CD colon n=3<br>Inflamed CD ileum n=3<br>Non-inflamed CD ileum n=3	Processed raw data (log2 transformed) were downloaded from ArrayExpress and values for *CD274* identified with Probe ID 223834_at. Manual calculation of FC and p value. All colon samples (n=6) displayed the exact same intensity value leading to a linear FC of 1.0 while no student t test could be performed.
11	E-MTAB-184<br>(ref. 2, 4, 7, 11)	Adult subjects<br><br>Controls:<br>Diagnostic colonoscopy due to symptoms unrelated to IBD. Patients were only included as normal controls after all clinically indicated examinations had concluded no signs of gastrointestinal disease.<br><br>Patients (n=23):<br>Patient samples were assigned to groups 'on treatment' or 'no treatment' according to Suppl. Table 1 from reference 4. Samples were further divided into 'inflamed' and	Endoscopic pinch biopsies from hepatic flexure (health and non-inflamed CD) or inflamed mucosa taken from the colon. Assessed as diseased or normal based on endoscopic findings at time of collection with final diagnosis by histo-pathological evaluation of H–E stained sections.	Illumina Human HT-12 arrays<br><br>Array design was downloaded from ArrayExpress (A-MEXP-1171) and *CD274* hybridization reference was identified with the gene name CD274.<br><br>Probe ID (hybridization reference):	Control n=20<br><br>On treatment:<br>Inflamed CD colon n=3<br>Non-inflamed CD colon n=11<br><br>No treatment:<br>Inflamed CD colon n=3<br>Non-inflamed CD colon n=8	Processed raw data (linear) were downloaded from ArrayExpress and values for *CD274* identified with hybridization reference ILMN_1701914. Manual calculation of FC and p value.

		‘non-inflamed’ based on sample information of disease state provided on ArrayExpress. One sample (ID 154) was excluded from analysis since it was defined as CD in the Suppl. Table 1 but as UC in the sample information on ArrayExpress. The dataset on ArrayExpress contained many more samples which were not included in our *CD274* analysis as they were not mentioned in the publications and did not include any information on medication.		ILMN_1701914		

Supplementary Table 4:

Details of patients from whom ileal tissue specimens were analysed.

Patient Group	Gender	Age	Site/Diagnosis
Cases (Crohn's Disease)	Female	19	Ileal
	Female	28	Ileal and colonic
	Male	33	Colonic[a]
	Male	64	Ileocecal
	Female	50	Ileal and colonic
	Female	29	Ileal
Control (Intestinal Tumour)	Female	56	Adenocarcinoma
	Female	61	Adenocarcinoma
	Female	66	Adenocarcinoma
	Male	76	Carcinoid tumour
	Male	46	Malignant melanoma
	Female	77	Carcinoid tumour
	Female	65	Carcinoid tumour
Control (Ulcerative Colitis)	Female	42	Ulcerative Colitis
	Male	43	Ulcerative Colitis

[a] Ileal involvement microscopically
